# Acute psychological effects of Coronavirus Disease 2019 outbreak among healthcare workers in China: a cross-sectional study

**DOI:** 10.1038/s41398-020-01031-w

**Published:** 2020-10-13

**Authors:** Ying Wang, Simeng Ma, Can Yang, Zhongxiang Cai, Shaohua Hu, Bin Zhang, Shiming Tang, Hanping Bai, Xin Guo, Jiang Wu, Hui Du, Lijun Kang, Huawei Tan, Ruiting Li, Lihua Yao, Gaohua Wang, Zhongchun Liu

**Affiliations:** 1 Department of Psychiatry, Renmin Hospital of Wuhan University, 430060 Wuhan, China; 2 Department of Psychiatry, First Affiliated Hospital, School of Medicine, Zhejiang University, 310003 Hangzhou, China; 3 Department of Psychiatry, Nanfang Hospital, Southern Medical University, 510515 Guangzhou, China; 4 Department of Psychosis Studies, Institute of Psychiatry, Psychology & Neuroscience, King’s College London, London, WC2R 2LS UK; 5 Department of Psychiatry, Wuhan Youfu Hospital, 430030 Wuhan, China; 6 Department of Psychiatry, Jing Men No. 2 People’s Hospital, 448000 Jingmen, China

**Keywords:** Psychiatric disorders, Diseases

## Abstract

To study the acute psychological effects of Coronavirus Disease 2019 (COVID-19) outbreak among healthcare workers (HCWs) in China, a cross-sectional survey was conducted among HCWs during the early period of COVID-19 outbreak. The acute psychological effects including symptoms of depression, anxiety, and post-traumatic stress disorder (PTSD) were assessed using the Patient Health Questionnaire-9 (PHQ-9), the Generalized Anxiety Disorder (GAD-7) questionnaire, and the Impact of Event Scale-Revised (IES-R). The prevalence of depression, anxiety, and PTSD was estimated at 15.0%, 27.1%, and 9.8%, respectively. Having an intermediate technical title, working at the frontline, receiving insufficient training for protection, and lacking confidence in protection measures were significantly associated with increased risk for depression and anxiety. Being a nurse, having an intermediate technical title, working at the frontline, and lacking confidence in protection measures were risk factors for PTSD. Meanwhile, not worrying about infection was a protective factor for developing depression, anxiety, and PTSD. Psychological interventions should be implemented among HCWs during the COVID-19 outbreak to reduce acute psychological effects and prevent long-term psychological comorbidities. Meanwhile, HCWs should be well trained and well protected before their frontline exposure.

## Introduction

Infectious diseases are one of the biggest threats to human beings. In December 2019, a novel coronavirus-infected disease^1–3^, now named as the Coronavirus Disease 2019 (COVID-19) by the World Health Organization, occurred in Wuhan, Hubei Province, China, and rapidly become a global health emergency^4^. Within 1 month, the number of confirmed COVID-19 cases, since it was first publicly reported^5^, has exceeded the total number of confirmed severe acute respiratory syndrome (SARS) cases in 2003 in China. As of April 2, 2020, 81,620 confirmed COVID-19 cases and 3322 deaths has been reported by the National Health Commission of China^6^. The outbreak of COVID-19 has caused public panic and triggered psychological stress^7^.

Healthcare workers (HCWs), the key personnel for controlling and eliminating the outbreak of a severe infectious disease, are at high risk of infection. During the outbreak of SARS, Ebola virus disease, and the Middle East respiratory syndrome (MERS), hundreds of HCWs were infected and even died^8–10^. Due to the fear of being infected or death, HCWs can experience various acute psychological effects in the early period of an outbreak^11^, including symptoms of depression, anxiety, and post-traumatic stress disorder (PTSD)^7^. Meanwhile, they can also experience isolation from their families or community because of infection transmission risk and stigmatization^11^.

Since the outbreak of COVID-19, a large sum of HCWs in China immediately devoted themselves to the fight against COVID-19, and more HCWs are involved or prepared, as the virus rapidly transmitted during the Chinese Spring Festival travel season. Facing an emergency, HCWs can suffer from acute psychological effects. Moreover, the unpredictable future of this epidemic, large burden in the clinical treatment and care, and shortage of medical protective resources in the initial period of the outbreak may aggravate the acute psychological effects among HCWs.

To alleviate the acute psychological effects of HCWs, a psychological intervention program has been launched in China^12^. However, the lack of baseline data and the unexplored risk factors for the psychological well-being of HCWs may mislead the direction and limit the effect of psychological intervention. Furthermore, previous studies mainly focused on the long-term psychological comorbidities of an outbreak, and the acute psychological effects among HCWs have been studied less. Accordingly, we performed this cross-sectional study to assess the acute psychological effects experienced by HCWs during the early period of the COVID-19 outbreak in China. We mainly assessed the symptoms of depression, anxiety, and PTSD, and explored the related risk factors among HCWs. The findings of this study may provide crucial evidence for psychological intervention, as well as possibilities for further comparisons in future studies.

## Subjects and methods

### Study design and participants

**Fig. 1:**
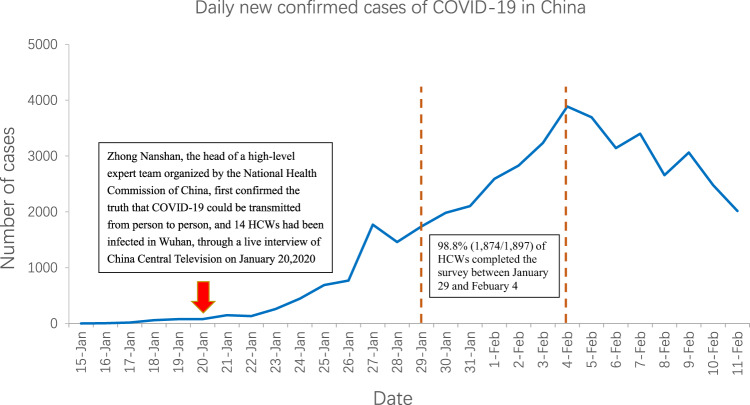
A time-trend diagram of the daily new confirmed cases of COVID-19 in China from January 15, 2020 to February 11, 2020. Data were obtained from the National Health Commission of China (Accessed on February 15, 2020, Available at: http://www.nhc.gov.cn/xcs/xxgzbd/gzbd_index.shtml).

This was a survey-based cross-sectional study performed from January 29, 2020 to February 7, 2020 in China. The actual time during which we conducted this study is presented in Fig. 1. The target population was doctors or nurses working in hospitals that established fever clinics or wards for patients with COVID-19. HCWs were mainly recruited from Wuhan (the capital of Hubei Province), the epicenter city of COVID-19 in China. For comparison, HCWs were also recruited from other regions within Hubei Province (except Wuhan) and from other provinces in China. Wenjuanxing (www.wjx.cn), an anonymous online survey tool used in a previous study^13^, was employed to collect data. The sample size was computed via the formula^14^ N = Z_α_^2^P (1 − P)/d^2^, where α = 0.05 and Zα = 1.96. The estimated acceptable margin of error for proportion d was 0.1, and the prevalence (P) of respondents with psychological distress was estimated at 20%^15^. Finally, the minimum sample size was estimated at about 1600.

This study was reviewed and approved by the Clinical Research Ethics Committee of Renmin Hospital of Wuhan University (WDRY2020-K004). Informed consent was obtained from all subjects prior to their enrollment by electronic way. Two options “yes/no” (whether subjects were willing to participate in the survey), were on the informed consent page, and only those who chose “yes” were taken to the questionnaire page. The anonymous survey had 74 required questions and took about 10 min. Respondents could terminate the survey at any time or during any question if they needed to. Participants or members of the public were not involved in the design, conduction, reporting, or dissemination plans of the study.

### Outcome and covariates

Depression was assessed using the Patient Health Questionnaire-9 (PHQ-9)^16^. The instrument had been validated in the Chinese population and showed good reliability (Cronbach’s α = 0.89)^17^. The PHQ-9 included nine items, the total score ranged from 0 to 27, and a score of 10 or greater was defined as depression. Anxiety was measured using the seven-item Generalized Anxiety Disorder (GAD-7) questionnaire^18^. The GAD-7 has been found to have good reliability in the Chinese population (Cronbach’s coefficient of 0.89)^19^. The total score of the GAD-7 ranged from 0 to 21, and a score of greater than 6 was classified as anxiety. PTSD was assessed using the Impact of Event Scale-Revised (IES-R)^20^. It consisted of 22 items and included three subscales: intrusion, avoidance, and hyperarousal. The total IES-R score was calculated as the mean response across all items, while subscale scores were generated from the mean response across all items within the specific subscale. A cutoff mean score of 2 or higher indicated obvious distress for the total and subscale scores^21^. The IES-R had been validated in the Chinese populations; the Cronbach’s alpha coefficients for subscales were three subscales were 0.89 (Intrusion), 0.85 (Avoidance), and 0.83 (Hyperarousal), respectively^22^.

Occupational factors included occupation (doctor/nurse), technical title (junior, intermediate, and senior), and type of hospital (secondary/tertiary). Other occupational factors related to COVID-19 and nosocomial infection were acquired by the following questions, and the previous name (2019-nCoV) of COVID-19 was used in the survey:

1. Working position: Are you directly engaged in the diagnosis and treatment or nursing of patients with fever or 2019-nCoV pneumonia (yes/no)? Those who responded with “yes” or “no” were defined as frontline or second-line HCWs.
2. Enough training for protection: Do you think that you have received enough training on prevention of nosocomial infection for 2019-nCoV pneumonia (yes/no)?
3. Enough resources for protection: Do you have enough resources to be protected according to the latest guidelines for the prevention and control of 2019-nCoV nosocomial infection (yes, no)?
4. Confidence in protection measures: Do you think the latest guidelines for the prevention and control of 2019-nCoV nosocomial infection can protect you from infection (yes/no)?
5. Worry about infection: Do you worry about your vulnerability to infection (yes/no)?

Socioeconomic factors were: gender (male or female), age (years), marital status (unmarried, married, widowed, or devoiced), educational level (undergraduate or less, postgraduate, or more), and location (Wuhan, Hubei Province (except Wuhan), or other provinces).

### Statistical analysis

Initially, 2367 completed questionnaires were received, with a response rate of 73.6%. The duration (seconds) to complete the questionnaire was recorded by Wenjuanxing. We excluded 346 questionnaires with a completion duration of less than 5 min (300 s) and more than 20 min (1200 s), to ensure that all questions had been well understood and completed consecutively by respondents. We further excluded 124 questionnaires from provinces that had recruited less than 20 participants. Totally, 470 questionnaires were excluded, leaving 1897 questionnaires for analysis. Respondents completing the included questionnaires and excluded questionnaires were comparable in age and sex.

Categorical data were presented as numbers (n)/percentage (%). The Chi-square test was used to compare the prevalence of depression, anxiety and PTSD between groups. Univariate regression analysis was conducted to determine whether any socioeconomic and occupational factors were associated with depression, anxiety, and PTSD. Binary logistic regression analysis was performed to examine the effects of occupational factors on depression, anxiety, and PTSD. The associations between potential risk factors and outcomes were presented as odds ratios (ORs) and 95% confidence intervals (CIs). Data analysis was conducted using SPSS version 20.0 (IBM Corp., Armonk, New York, United States). The significance level was set at α = 0.05.

## Results

The 1897 HCWs consisted of 332 males (17.5%) and 1 565 females (82.5%). Most of the HCWs were aged 25–40 years (61.7%), married (67.5%), and had an educational level of undergraduate or less (84.8%). Nearly half of the HCWs (49.5%) were recruited from hospitals in Wuhan, 29.7% were doctors, 70.3% were nurses, 76.3% worked in tertiary hospitals, and 39.1% worked at the frontline against COVID-19. The majority of the HCWs had received sufficient training (71.0%) or had enough resources (78.8%) for protection, had confidence in the protection measure (68.4%), and worried about being infected (82.8%) (Table 1).

**Table 1:** Characteristics of the healthcare workers of this study.

Variables	Number	Percentage (%)
Total	1897	100.0
*Socioeconomic factors*		
Gender		
Male	332	17.5
Female	1565	82.5
Age, years		
18–25	327	17.2
–30	576	30.4
–40	593	31.3
>40	401	21.1
Marital status		
Unmarried	617	32.5
Married^a^	1280	67.5
Education level		
Undergraduate or less	1608	84.8
Postgraduate or higher	289	15.2
Location		
Wuhan	939	49.5
Hubei except Wuhan	505	26.6
Other provinces	453	23.9
*Occupational factors*		
Occupation		
Doctor	563	29.7
Nurse	1334	70.3
Technical title		
Junior	1113	58.7
Intermediate	532	28.0
Senior	252	13.3
Type of hospital		
Tertiary	1447	76.3
Secondary	450	23.7
Working position		
Frontline	742	39.1
Second-line	1155	60.9
Enough training for protection	1347	71.0
Enough resources for protection	1494	78.8
Have confidence in protection measures	1297	68.4
Worry about infection	1570	82.8

^a^Including 17 widowed or divorced healthcare workers.

Overall, the prevalence of depression, anxiety, and PTSD was assessed at 15.0%, 27.1%, and 9.8%, respectively. The prevalence of depression, anxiety, and PTSD among HCWs working at the frontline was assessed at 21.7%, 38.5%, and 15.4%, respectively. A significantly higher prevalence of depression, anxiety, and PTSD was observed in HCWs that were females, working in Wuhan, working at the frontline, received insufficient training and recourses for protection, lack of confidence in protection measures, and worried about being infected (all *p* < 0.05). Nurses and those with an intermediate technical title had high prevalence of anxiety and PTSD (all *p* < 0.05). No statistical differences were detected among HCWs in different age groups, marital status, education level, and type of hospital (all *p* > 0.05) (Table 2).

**Table 2:** Prevalence of acute psychological effects among healthcare workers.

Variables	Total	Depression			Anxiety			PTSD		
		Yes	No	*p* value	Yes	No	*p* value	Yes	No	*p* value
Total	1897	285 (15.0)	1612 (85.0)	NA	515 (27.1)	1382 (72.9)	NA	185 (9.8)	1712 (90.2)	NA
*Socioeconomic factors*										
Gender										
Male	332	36 (10.8)	296 (89.2)	0.024	71 (21.4)	261 (78.6)	0.011	21 (6.3)	311 (93.7)	0.027
Female	1565	249 (15.9)	1316 (84.1)		444 (28.4)	1121 (71.6)		164 (10.5)	1401 (89.5)	
Age, years										
18–25	327	53 (16.2)	274 (83.8)	0.260	92 (28.1)	235 (71.9)	0.149	35 (10.7)	292 (89.3)	0.489
–30	576	81 (14.1)	495 (85.9)		142 (24.7)	434 (75.3)		47 (8.2)	529 (91.8)	
–40	593	100 (16.9)	493 (83.1)		179 (30.2)	414 (69.8)		61 (10.3)	532 (89.7)	
>40	401	51 (12.7)	350 (87.3)		102 (25.4)	299 (74.6)		42 (10.5)	359 (89.5)	
Marital status^a^										
Unmarried	617	100 (16.2)	517 (83.8)	0.351	160 (25.9)	457 (74.1)	0.440	56 (9.1)	561 (90.9)	0.544
Married^a^	1280	185 (14.5)	517 (40.4)		355 (27.7)	925 (72.3)		129 (10.1)	1151 (89.9)	
Education level										
Undergraduate or less	1608	241 (15.0)	1367 (85.0)	0.988	431 (26.8)	1177 (73.2)	0.469	163 (10.1)	1445 (89.9)	0.221
Postgraduate or higher	289	44 (15.2)	245 (84.8)		84 (29.1)	205 (70.9)		22 (7.6)	267 (92.4)	
Location										
Wuhan	939	161 (17.1)	778 (82.9)	0.012	311 (33.1)	628 (66.9)	<0.001	116 (12.4)	823 (87.6)	<0.001
Hubei except Wuhan	505	57 (11.3)	448 (88.7)		99 (19.6)	406 (80.4)		37 (7.3)	468 (92.7)	
Other provinces	453	67 (14.8)	386 (85.2)		105 (23.2)	348 (76.8)		32 (7.1)	421 (92.9)	
*Occupational factors*										
Occupation										
Doctor	563	77 (13.7)	486 (86.3)	0.319	133 (23.6)	430 (76.4)	0.029	39 (6.9)	524 (93.1)	0.009
Nurse	1334	208 (15.6)	1126 (84.4)		382 (28.6)	952 (71.4)		146 (10.9)	1188 (89.1)	
Technical title										
Junior	1113	164 (14.7)	949 (85.3)	0.061	291 (26.1)	822 (73.9)	0.004	100 (9.0)	1013 (91.0)	0.023
Intermediate	532	93 (17.5)	439 (82.5)		170 (32.0)	362 (68.0)		67 (12.6)	465 (87.4)	
Senior	252	28 (11.1)	224 (88.9)		54 (21.4)	198 (78.6)		18 (7.1)	234 (92.9)	
Type of hospital										
Tertiary	1447	218 (15.1)	1229 (84.9)	0.987	396 (27.4)	1051 (72.6)	0.746	148 (10.2)	1299 (89.8)	0.245
Secondary	450	67 (14.9)	383 (85.1)		119 (26.4)	331 (73.6)		37 (8.2)	413 (91.8)	
Working position										
Frontline	742	161 (21.7)	581 (78.3)	<0.001	286 (38.5)	456 (61.5)	<0.001	114 (15.4)	628 (84.6)	<0.001
Second-line	1155	124 (10.7)	1031 (89.3)		229 (19.8)	926 (80.2)		71 (6.1)	1084 (93.9)	
Enough training for protection										
Yes	1347	167 (12.4)	1180 (87.6)	<0.001	323 (24.0)	1024 (76.0)	<0.001	115 (8.5)	1232 (91.5)	0.007
No	550	118 (21.5)	432 (78.5)		192 (34.9)	358 (65.1)		70 (12.7)	480 (87.3)	
Enough resources for protection										
Yes	1494	203 (13.6)	1291 (86.4)	0.001	377 (25.2)	1117 (74.8)	<0.001	138 (9.2)	1356 (90.8)	0.173
No	403	82 (20.3)	321 (79.7)		138 (34.2)	265 (65.8)		47 (11.7)	356 (88.3)	
Confidence in protection measures										
Yes	1297	152 (11.7)	1145 (88.3)	<0.001	301 (23.2)	996 (76.8)	<0.001	99 (7.6)	1198 (92.4)	<0.001
No	600	133 (22.2)	467 (77.8)		214 (35.7)	386 (64.3)		86 (14.3)	514 (85.7)	
Worry about infection										
Yes	1570	273 (17.4)	1297 (82.6)	<0.001	497 (31.7)	1073 (68.3)	<0.001	184 (11.7)	1386 (88.3)	<0.001
No	327	12 (3.7)	315 (96.3)		18 (5.5)	309 (94.5)		1 (0.3)	326 (99.7)	

*NA* not available, *PTSD* post-traumatic stress disorder.

^a^Including 17 widowed or divorced healthcare workers.

Results of univariate logistic regression are presented in Table 3. Females, frontline HCWs, those who worked in Wuhan, had insufficient training and resources for protection, lacked confidence in protection measures, and worried about being infected were more likely to be afflicted with depression, anxiety, and PTSD. Nurses (vs. doctors) or those with an intermediate technical title (vs. junior technical title) were at higher risk of anxiety and PTSD.

**Table 3:** Association of socioeconomic and occupational factors and acute psychological effects among healthcare workers.

Variables	Total	Depression			Anxiety			PTSD		
		*n*	OR (95% CI)	*p* value	*n*	OR (95% CI)	*p* value	*n*	OR (95% CI)	*p* value
*Socioeconomic factors*										
Gender										
Male	332	36	Reference		71	Reference		21	Reference	
Female	1565	249	1.56 (1.07, 2.26)	0.020	444	1.46 (1.10, 1.94)	0.010	164	1.73 (1.08, 2.78)	0.022
Age, years										
18–25	327	53	Reference		92	Reference		35	Reference	
–30	576	81	0.85 (0.58, 1.23)	0.384	142	0.84 (0.62, 1.14)	0.251	47	0.74 (0.47, 1.18)	0.202
–40	593	100	1.05 (0.73, 1.51)	0.798	179	1.10 (0.82, 1.49)	0.514	61	0.96 (0.62, 1.48)	0.843
>40	401	51	0.75 (0.50, 1.14)	0.182	102	0.87 (0.63, 1.21)	0.413	42	0.98 (0.61, 1.57)	0.920
Marital status										
Unmarried	617	100	Reference		160	Reference		56	Reference	
Married^a^	1280	185	0.87 (0.67, 1.14)	0.317	355	1.10 (0.88, 1.36)	0.408	129	1.12 (0.81, 1.56)	0.491
Education level										
Undergraduate or less	1608	241	Reference		431	Reference		163	Reference	
Postgraduate or higher	289	44	1.02 (0.72, 1.44)	0.917	84	1.12 (0.85, 1.48)	0.426	22	0.73 (0.46, 1.16)	0.185
Location										
Wuhan	939	161	Reference		311	Reference		116	Reference	
Hubei except Wuhan	505	57	0.62 (0.45, 0.85)	0.003	99	0.49 (0.38, 0.64)	<0.001	37	0.56 (0.38, 0.83)	0.003
Other provinces	453	67	0.84 (0.62, 1.14)	0.266	105	0.61 (0.47, 0.79)	<0.001	32	0.54 (0.36, 0.81)	0.003
*Occupational factors*										
Occupation										
Doctor	563	77	Reference		133	Reference		39	Reference	
Nurse	1334	208	1.17 (0.88, 1.55)	0.286	382	1.30 (1.03, 1.63)	0.025	146	1.65 (1.14, 2.39)	0.008
Technical title										
Junior	1113	164	Reference		291	Reference		100	Reference	
Intermediate	532	93	1.23 (0.93, 1.62)	0.152	170	1.33 (1.06, 1.66)	0.014	67	1.46 (1.05, 2.03)	0.024
Senior	252	28	0.72 (0.47, 1.11)	0.137	54	0.77 (0.55, 1.07)	0.120	18	0.78 (0.46, 1.31)	0.349
Type of hospital										
Tertiary	1447	218	Reference		396	Reference		148	Reference	
Secondary	450	67	0.99 (0.73, 1.33)	0.927	119	0.95 (0.75, 1.21)	0.701	37	0.79 (0.54, 1.15)	0.211
Working position										
Second-line	1155	124	Reference		229	Reference		71	Reference	
Frontline	742	161	2.43 (1.83, 3.22)	<0.001	286	2.54 (2.06, 3.12)	<0.001	114	2.77 (2.03, 3.79)	<0.001
Enough training for protection										
Yes	1347	167	Reference		323	Reference		115	Reference	
No	550	118	1.93 (1.49, 2.50)	<0.001	192	1.70 (1.37, 2.11)	<0.001	70	1.56 (1.14, 2.14)	0.006
Enough resources for protection										
Yes	1494	203	Reference		377	Reference		138	Reference	
No	403	82	1.63 (1.22, 2.16)	0.001	138	1.54 (1.22, 1.96)	<0.001	47	1.30 (0.91, 1.84)	0.146
Confidence in protection measures										
Yes	1297	152	Reference		301	Reference		99	Reference	
No	600	133	2.15 (1.66, 2.77)	<0.001	214	1.84 (1.49, 2.27)	<0.001	86	2.03 (1.49, 2.75)	<0.001
Worry about infection										
Yes	1570	273	Reference		497	Reference		184	Reference	
No	327	12	0.18 (0.10, 0.33)	<0.001	18	0.13 (0.08, 0.21)	<0.001	1	0.02 (0.003, 0.17)	<0.001

*OR* odds ratio, *CI* confidence interval, *PTSD* post-traumatic stress disorder.

^a^Including 17 widowed or divorced healthcare workers.

The results of the binary logistic regression analysis are listed in Table 4. Controlling for potential confounding variables, intermediate technical title, working at the frontline, insufficient training for protection, and a lack of confidence in protection measures were significantly associated with an increased risk of depression and anxiety. Being a nurse, having an intermediate technical title, working at the frontline, and a lack of confidence in protection measures were risk factors for PTSD. Worry about infection was a risk factor for developing depression, anxiety, and PTSD. HCWs working in Hubei Province (except Wuhan) had a lower risk for anxiety than those who worked in Wuhan.

**Table 4:** Risk factors of acute psychological effects among healthcare workers identified by binary logistic regression analysis.

Variables	Depression		Anxiety		PTSD	
	OR (95% CI)	*p* value	OR (95% CI)	*p* value	OR (95% CI)	*p* value
Gender (Ref: male)						
Female	1.43 (0.94, 2.18)	0.097	1.24 (0.89, 1.74)	0.210	1.33 (0.78, 2.28)	0.296
Location ((Ref: Wuhan)						
Hubei except Wuhan	0.73 (0.52, 1.03)	0.075	0.58 (0.44, 0.77)	<0.001	0.71 (0.47, 1.08)	0.113
Other provinces	1.26 (0.88, 1.80)	0.205	0.91 (0.67, 1.22)	0.524	0.88 (0.56, 1.40)	0.593
Occupation (Ref: doctor)						
Nurse	1.06 (0.73, 1.54)	0.746	1.24 (0.91, 1.69)	0.184	1.62 (1.01, 2.62)	0.048
Technical title (Ref: junior)						
Intermediate	1.39 (1.03, 1.88)	0.032	1.61 (1.25, 2.07)	<0.001	1.88 (1.32, 2.67)	<0.001
Senior	1.03 (0.63, 1.69)	0.913	1.33 (0.89, 1.98)	0.166	1.67 (0.91, 3.06)	0.100
Working position (Ref: second-line)						
Frontline	2.00 (1.53, 2.63)	<0.001	2.07 (1.66, 2.59)	<0.001	2.27 (1.63, 3.17)	<0.001
Enough training for protection (Ref: yes)						
No	1.50 (1.11, 2.00)	0.008	1.31 (1.03, 1.68)	0.031	1.20 (0.84, 1.71)	0.323
Enough resources for protection (Ref: yes)						
No	1.12 (0.81, 1.55)	0.501	1.20 (0.91, 1.58)	0.203	0.95 (0.64, 1.42)	0.817
Confidence in protection measures (Ref: yes)						
No	1.71 (1.28, 2.27)	<0.001	1.47 (1.16, 1.87)	0.002	1.73 (1.23, 2.43)	0.002
Worry about infection (Ref: yes)						
No	0.25 (0.14, 0.46)	<0.001	0.17 (0.10, 0.27)	<0.001	0.03 (0.01, 0.23)	0.001

*OR* odds ratio, *CI* confidence interval, *PTSD* post-traumatic stress disorder, *Ref* reference.

## Discussion

### Main findings

This cross-sectional study, based on 1897 participants assessed the acute psychological effects of the COVID-19 outbreak among HCWs in China. The prevalence of depression, anxiety, and PTSD in HCWs in the first month of COVID-19 outbreak was 15.0%, 27.1%, and 9.8%, respectively. Overall, females, and those who worked in Wuhan, worked at the frontline, had insufficient training and recourses for protection, lacked confidence in protection measures, and worried about being infected had a significantly higher prevalence of psychological effects. Generally, having an intermediate technical title, working at the frontline, insufficient training for protection, a lack of confidence in protection measures, and worry about infection were risk factors for depression, anxiety, and PTSD. In comparison with a recent study involving 1257 HCWs in China that identified factors associated with mental health outcomes among HCWs exposed to the COIVD-19^23^, our study firstly explored the association of several occupational factors with risk of acute psychological effects of the COVID-19 outbreak among HCWs with a larger sample size.

HCWs were at high risk of exposure to COVID-19, and were faced with increased levels of stress. In response to actual or possible threat, stress enhance the possibility of forming trauma-related memories^24^, and is a defining feature of PTSD^25^. Moreover, stress plays a critical role in the development and expression of many other psychiatric disorders^25^. Several pathways exist to establish the link between stress and psychiatric disorders, for instance, inflammation. There is evidence that psychological stress can trigger significant increases in inflammatory activity^26^. Notably, elevated concentrations of inflammatory signals, including cytokines and C-reactive protein, have been reported in patients with PTSD, generalized anxiety disorder, and depression^27,28^. However, because the pathophysiological mechanism is very complex, it is likely that many pathways act simultaneously to contribute to the psychiatric disorders.

### Comparison with other studies

In our study, the prevalence of depression, anxiety, and PTSD was estimated at 15.0%, 27.1%, and 9.8%, respectively. The psychological effects of the COVID-19 outbreak seem to be less severe among HCWs than those of the SARS outbreak. According to a systematic review based on thirty-two studies involving 26,869 participants^15^, the estimated average rate of depression, anxiety, and PTSD during outbreaks of infectious disease (mainly SARS) was ~46% (ranging from 23^29^ to 74%^30^), 45% (ranging from 19^31^ to 77%^30^), and 21% (ranging from 10^32^ to 33%^33^), respectively. This difference between our study and previous ones may be attributed to the variations in measurement tools. For example, the Center for Epidemiologic Studies Depression Scale (CES-D) was used by Liu et al.^29^ while the Chinese Health Questionnaire (CHQ) was chose by Chong et al.^30^ for the assessment of depression, both of which differed from this study. Another possible explanation is that our study was conducted at the early stage of the outbreak, while studies regarding SARS were generally conducted at the late stage^30^ of the outbreak or after the outbreak had discharged^31^. The differences might reflect variations between the acute and long-term psychological effects triggered by an outbreak, and the prevalence of psychological stress among HCWs may increase after the initial period of an outbreak.

We found that HCWs with an intermediate technical title were at higher risk of anxiety and PTSD. One possible explanation was that HCWs with an intermediate technical title may be burdened with more work responsibility than those with a junior technical title, as well as longer work time in the wards than HCWs with a senior technical title. As a result, they may be associated with higher risk of psychological distress. In a previous study^30^, being both male and female gender were associated with being at risk for PTSD and anxiety, respectively. However, being female was associated with an increased risk for depression, anxiety, and PTSD only in our initial analysis. After controlling for confounders, the association was dismissed.

Another finding in our study was that working at the frontline was an independent risk factor for depression, anxiety, and PTSD, which has also been demonstrated by previous studies during the outbreak of severe acute respiratory syndrome (SARS) in 2003^29,30,34–36^. It was indicated that HCWs working at the frontline should be the priority to receive psychological assistance. We also found that factors related to nosocomial infection, including insufficient training and recourses for protection, lack of confidence in protection measures, and worried about being infected, were generally found to be associated with psychological stress. Among these factors, previous studies indicated that lack of confidence in protection measures was a risk factor for anxiety^37^ while sufficient training protected against the development of anxiety^31^. This highlights the importance of essential training and good protection for preventing nosocomial infection in HCWs before they are faced with COVID-19 directly, to reduce the acute psychological effects.

### Strengths and limitations

The primary strength of this study is the timing. We initiated this study when the confirmed cases of COVID-19 were increased rapidly and the narrow time period during which all of the participants completed the survey, with 98.8% of them completing it within 7 days (from January 29 to February 4, 2020) reflected the psychological effects of the COVID-19 outbreak among HCWs could be well. Second, we used THE PHQ-9, GAD-7, and IES-R to assess depression, anxiety, and PTSD, which have been validated in the Chinese population and show good reliability^17,19,22^. The three brief tools together consist of only 38 single-choice questions. The timesaving advantage makes them more acceptable for respondents to complete thoroughly, especially for the frontline HCWs. Another strength is that we had a larger sample size compared with previous studies that focused on the SARS outbreak ^15^. Thus, the results of the present study are robust and reliable.

The limitations of this study should also be acknowledged. First, the present study could not establish a causal relationship between potential risk factors and the acute psychological effects among HWCs because of the cross-sectional study design. Second, there is the possibility of selection bias, as the second-line HCWs tended to have more time and interest in participation, and thus the prevalence of acute psychological effects could be underestimated. However, because the study was conducted anonymously through an online tool with a response rate of 73.6%, information of those who declined to respond could not be collected, we therefore failed to assess the extent of potential selection bias. Third, HCWs may experience more psychological distress compared with the general population even without an outbreak of an infectious disease, and the status of psychological distress may vary overtime, whether HCWs suffered from more severe psychological burden during early exposure to COVID-19 than that before or after the COVID-19 epidemic remained unknown because of the lack of baseline and follow-up data.

### Conclusions and policy implications

A proportion of HCWs suffer from acute psychological effects caused by the COVID-19 outbreak. High-risk HCWs for acute psychological effects were those with an intermediate technical title, working at the frontline, lacking training for protection, lacking confidence in protection measures, and worrying about being infected. Psychological interventions should be implemented among HCWs during the COVID-19 outbreak to reduce acute psychological effects and prevent long-term psychological comorbidities. Meanwhile, HCWs should be well trained and well protected before their frontline exposure.

## Supplementary information

STROBE_checklist
